# Clinical Management and Epidemiology of Scorpion Stings in Khuzestan Province, Iran: A Five-year Study: Five-year Study of Scorpion Stings in Khuzestan Province

**DOI:** 10.31661/gmj.vi.3810

**Published:** 2025-06-03

**Authors:** Soheila Aminzadeh, Ali Sahnalizadeh, Mohammad Hossein Gharibreza, Elham Farhadi, Ali Hasan Rahmani, Maryam Salehcheh

**Affiliations:** ^1^ Toxicology Research Center, Medical Basic Sciences Research Institute, Ahvaz Jundishapur University of Medical Sciences, Ahvaz, Iran; ^2^ Student Research Committee, Ahvaz Jundishapur University of Medical Sciences, Ahvaz, Iran; ^3^ Department of Medicine, Faculty of Medicine, Jundishapur University of Medical Sciences, Ahvaz, Iran; ^4^ Department of Clinical Toxicology, Razi Hospital, School of Medicine, Ahvaz Jundishapur University of Medical Sciences, Ahvaz, Iran; ^5^ Clinical Research Development Unit, Golestan Hospital, Ahvaz Jundishapur University of Medical Sciences, Ahvaz, Iran; ^6^ Department of Toxicology, School of Pharmacy, Ahvaz Jundishapur University of Medical Sciences, Ahvaz, Iran

**Keywords:** Scorpion, Clinical Signs, Laboratory Signs

## Abstract

**Background:**

Scorpion stings are a significant public health concern in many countries, particularly in Iran’s Khuzestan province. This study investigates the clinical and laboratory correlations in patients hospitalized for scorpion stings at Razi Hospital, Ahvaz, Iran, from 2018 to 2022, aiming to enhance patient care and preventive strategies.

**Materials and Methods:**

This descriptive, cross-sectional, retrospective study employed census sampling. Age, gender, sting season, sting site, delay to visiting hospital, hospitalization duration, antiscorpion treatment vials prescription, clinical symptom and laboratory findings were collected from medical records of patients hospitalized for scorpion stings during the study period, using a standardized checklist for clinical and laboratory parameters.

**Results:**

Our analysis of 799 scorpion sting cases revealed a male predominance (55.9%) and the highest incidence among individuals aged 21–40 years, with most stings occurring in summer. The extremities, particularly hands and feet, were the most common sting sites. Pain, erythema, and swelling were the leading symptoms, with most patients seeking medical care within three hours. Hospitalization was common, typically lasting at least one day. Disturbances in urinalysis (U/A) were the most frequent laboratory abnormality. Younger women in intensive care exhibited severe symptoms, including seizures, jaundice, and hematuria, which correlated with abnormalities in CBC, biochemical markers, PT, and U/A. These findings highlight the importance of timely clinical and laboratory assessments to improve outcomes.

**Conclusion:**

Scorpion stings continue to represent a public health challenge with a range of clinical manifestations and laboratory correlations. By enhancing awareness and preparedness, we can mitigate the impact of this health concern and improve patient outcomes for those affected by scorpion envenomations.

## Introduction

Scorpion stings represent a critical public health issue with substantial medical, social, and economic ramifications, particularly in tropical and subtropical regions [1].​ This problem is especially pronounced in hotter climates, such as the Khuzestan province in southwestern Iran, where environmental conditions foster a higher prevalence of scorpion encounters [2][3].

Scorpions, venomous arachnids belonging to the class Arachnida and order Scorpiones, pose significant danger to human health. In Iran alone, a diverse array of 25 scorpion species has been identified, predominantly classified within five genera: Mesobuthus, Compsobuthus, Hottentotta (also known as Buthotus), Orthochirus, Androctonus, and Hemiscorpius. These genera include species that are commonly implicated in stings throughout the Khuzestan region [4].

Among these, Hemiscorpius lepturus is notably responsible for a disproportionate number of fatalities during the warmer months, despite accounting for only 12% of scorpion sting cases in the area. Alarmingly, this species is implicated in over 95% of all scorpion sting-related deaths in Iran, highlighting the severity of its toxicological impact [5][6][3]. The clinical effects of scorpion stings can vary dramatically, influenced by numerous factors such as the season, the specific species involved, the location and quantity of bites, the amount of venom injected, and patient-specific variables like age, weight, and pre-existing health conditions. Importantly, victims with a history of cardiopulmonary disease or allergic reactions may experience amplified clinical responses [7][8].

The range of symptoms following a scorpion sting is broad, encompassing both local and systemic effects. Localized reactions can manifest as pain, swelling, erythema, and necrosis, while systemic responses may include severe complications such as hemolysis, pulmonary edema, seizures, and renal failure, among others [6][1][3]. The medical response to these envenomations varies; some patients can be managed on an outpatient basis, others require hospitalization, and a subset may necessitate intensive care management. The treatment protocol for scorpion envenomation typically includes the use of fresh frozen plasma (FFP), packed red blood cells (P.C), platelet transfusions (PLT), and specific antiscorpion serum to mitigate the dangerous effects of the venom. The administration of antiscorpion serum, a targeted therapeutic approach, aims to neutralize the toxic impact of the venom. In Iran, the standard practice has been the use of a multivalent antivenom effective against six of the most common Iranian scorpions—a therapeutic strategy that has dominated scorpion sting management for over three decades [1][3].

In this study, we endeavor to shed light on the epidemiological data surrounding scorpion stings in Razi Hospital, Ahvaz, Iran, over a five-year period from 2018 to 2022. By documenting cases, we will offer insights into clinical complications, laboratory findings, treatment modalities, hospitalization rates, and overall mortality associated with scorpion stings in this high-risk region. Through this examination, we aim to enhance understanding and inform future strategies for managing this pressing public health concern.

## Materials and Methods

This study represents a descriptive, cross-sectional, and retrospective analysis conducted under the oversight of the Ethics Committee of the Research Council at Jundishapur University of Ahvaz (Ethic Code: IR.AJUMS.HGOLESTAN.REC.1402.083). The study population comprised all patients who experienced scorpion stings and visited Razi Hospital of Ahvaz during the years 2018 to 2022. A census sampling method was employed to include every eligible patient within this timeframe. Data were meticulously gathered using structured checklists [9] and questionnaires, which facilitated systematic comparison and assessment. The information collected encompassed crucial demographic data, including age and gender, enabling an understanding of the distribution of scorpion sting cases within different population segments. Additionally, clinical data including: disease symptoms, types of treatments administered—including PLT therapy, FFP, P.C, and Antiscorpium—as well as insights into patient admission profiles, outcomes including intubation or death, history of readmission, time delay until reaching the hospital, season and location of the bite, were compiled. Laboratory assessments were also integral to this study, involving the collection of test results for CBC, BIO, PT, U/A, and evaluations of organ function. Inclusion criteria were strictly defined, focusing on patients who had sustained scorpion stings, while exclusion criteria were limited to circumstances where patient records were inaccessible or when interviews could not be conducted. Patient follow-up was contingent upon their return for subsequent consultations. Specifically, patients who revisited the clinic during the five-year research period were re-interviewed, and their initial scorpion sting records were updated with any new information. Every participating patient or their guardians signed informed consent forms, ensuring that ethical considerations were upheld throughout the study. Data analysis was performed with a strong emphasis on maintaining the confidentiality of clinical information, ensuring that individual patient identities remained anonymous.

### Statistical Analysis

Statistical analysis of the collected data was performed using SPSS software, version 24 (SPSS Inc., Chicago, IL, USA). To assess the normality of the data distribution, both the Kolmogorov-Smirnov test and Q-Q plots were utilized. For quantitative variables, the central tendency was summarized using the mean and/or median, while the dispersion was characterized by the standard deviation and/or interquartile range. In the case of qualitative variables, frequency and percentage distributions were employed to effectively represent the data. Group comparisons were executed through independent Student’s t-tests. A significance level (P-value) of less than 0.05 was established as the threshold for statistical significance. Furthermore, relevant visualizations were produced utilizing Excel charts to enhance the presentation of the findings (Microsoft, USA).

### Ethical Approval

Every participating patient or their guardians signed informed consent forms, ensuring that ethical considerations were upheld throughout the study. This study represents a descriptive, cross-sectional, and retrospective analysis conducted under the oversight of the Golestan Hospital Research Ethics Committee (IR.AJUMS.HGOLESTAN.REC.1402.083).

## Results

**Figure-1:**
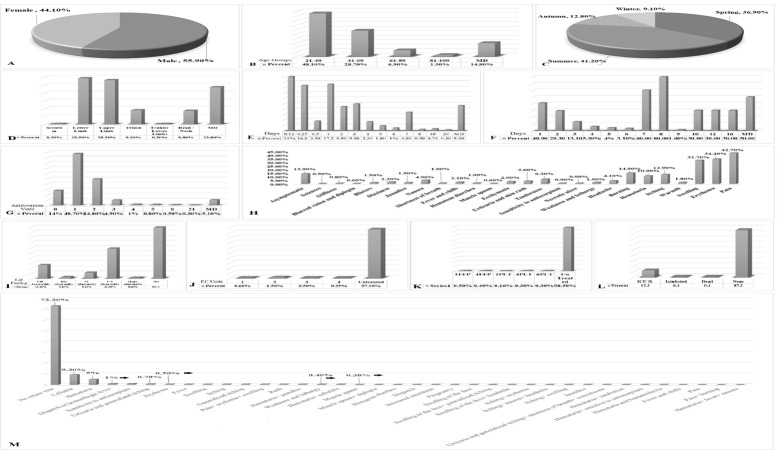
Figure 1. Demographic, Clinical and laboratory features of patients. Frequency of patients in different categories of: A. Gender, B. Age, C. Sting season, D. Sting site, E. Delay to visiting hospital, F. Hospitalization duration. G. Antiscorpion treatment vials prescription, H. clinical symptom of all patients, I: Laboratory findings, J: P.C unit prescription, K: FFP and PLT unit prescription, L: Prognose of patient, M: Clinical symptom in return visit. MD: Missed Data

**Figure-2:**
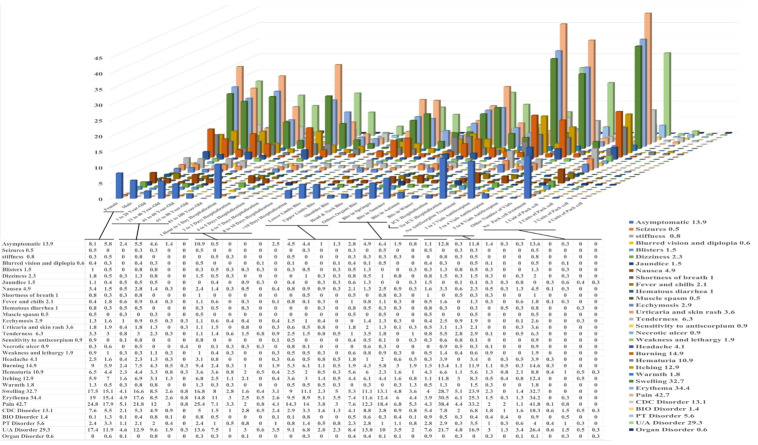
Figure 2. Overall view on clinical findings of patients in different categories of gender, age, sting season and site, hospitalization profile, treatment, and prognosis.

**Figure-3:**
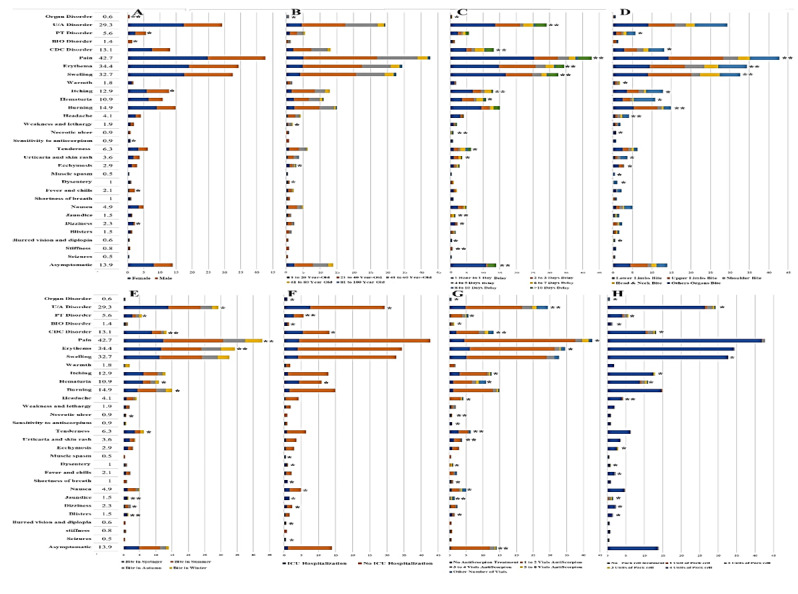
Figure 3. Detailed view on clinical findings of patients in different categories of gender (A), age (B), delay to visiting hospital (C), sting site (D), sting season (E), ICU admission history (F), antiscorpion treatment prescription (G), and P.C treatment prescription (H). *: P-value ≤ 0.01; **: P-value ≤0.001.

### Demographic Findings

This study comprised a total of 799 scorpion sting patients who visited Razi Hospital in Ahvaz between 2018 and 2022. These individuals were included based on the availability of their medical records or their ability to be interviewed. The mean age of the study population was 38.68±15.91 years. As illustrated in Figure-1A, among these patients, 447 (55.9%) were male, while 352 (44.1%) were female. The average age of the male patients was 36.47±16.07 years, compared to 38.64±15.73 years for female patients. The highest incidence of scorpion stings was observed in individuals aged 21 to 40 years, accounting for 384 cases (48.1%). This was followed by the 41 to 60-year age group, which comprised 299 cases (28.7%), as depicted in Figure-1B. Furthermore, the results indicated that the summer months experienced the greatest frequency of scorpion stings, with 329 cases (41.2%) recorded, including 134 males and 195 females. In contrast, the winter months recorded the lowest incidence, with only 73 cases (9.1%), as shown in Figure-1C.

### Clinical and Laboratory Findings

The clinical and laboratory findings from this study reveal significant insights into the patterns and consequences of scorpion stings. Based on the data regarding bite locations, as illustrated in Figure-1D, the majority of bites occurred in the lower limbs, with 236 cases (29.5%), followed closely by the upper limbs, which accounted for 226 cases (28.3%). Notably, 160 patients (20%) sought medical attention within the first three hours after the sting, while 137 patients (17.1%) presented to the hospital within the first day after the bite. These two groups exhibited the highest frequencies of hospital visits following the bite incident, as depicted in Figure-1E. As shown in Figure-1F, the peak hospitalization rates among the admitted patients were recorded on the first day, with a total of 327 individuals (40.9%), followed by 234 patients (29.3%) on the second day. Regarding treatment, the present study indicates that 389 patients (48.7%) received one vial of antiscorpion serum, while 198 patients (24.8%) were administered two vials.

These two subgroups represented the highest counts in the analysis of antiscorpion serum administration (Figure-1G). In terms of clinical symptoms observed, pain was reported as the most common symptom, affecting 341 patients (42.7%). This was followed by erythema in 275 patients (34.4%), swelling in 261 patients (32.7%), and burning sensations experienced by 119 patients (14.9%). Importantly, 111 individuals (13.9%) were identified as asymptomatic (Figure-1H). Laboratory evaluations revealed that U/A disturbance were noted in 234 cases (29.3%), while CBC disturbance was observed in 105 cases (13.1%), marking these as the most prevalent laboratory findings among those examined (Figure-1I).

Figure-1J illustrates that a significant majority of patients—776 (97.1%)—did not receive platelet cell (P.C) treatments. The highest rate of platelet administration recorded was two units, given to 12 patients (1.5%), while one unit was administered to five patients (0.6%). With regard to FFP treatments, usage statistics indicated that four patients (0.5%) received one unit of FFP, and three patients (0.4%) were treated with four units. Moreover, two patients (0.3%) received four units of platelets, and another two patients (0.3%) were administered six units. Notably, the remaining 787 patients (98.5%) did not receive either FFP or platelet treatments (Figure-1K). Among the total of 799 patients who presented for treatment, 98 patients (12.3%) required admission to the Intensive Care Unit (ICU), with two patients (0.3%) needing intubation and the same number died.

The remaining patients did not require hospitalization or intubation (Figure-1L). Follow-up data indicated that 213 patients (26.7%) returned for further assessment, of which 74 patients (9.3%) reported complaints of cellulitis, and 41 patients (5.1%) presented with hematuria (Figure-1M).

### Examining the Relationship between Clinical and Laboratory Symptoms and Other Variables

Figure-2 provides a comprehensive overview of the frequency of clinical and laboratory symptoms observed across various groups related to the investigated variables. These variables include gender, age, duration of hospitalization, site of the sting, season of the sting, ICU admission profiles, and the treatments administered (P.C and AntiScorpion). Statistical analyses were performed using the chi-square test and Fisher’s exact test to establish significant differences, as shown in Figure-2. The following discussion will detail key findings, emphasizing clinical data that includes symptoms such as the absence of symptoms, seizures, blisters, blurred vision, dizziness, jaundice, nausea, shortness of breath, fever and chills, bloody diarrhea, ecchymosis, necrotic ulcers, and hematuria.

A significance cutoff of P-value≤ 0.05 was employed. In exploring the relationship between these clinical symptoms and gender, it was identified that only dizziness, fever and chills, and sensitivity to the antiscorpion treatment demonstrated significant differences between male and female groups. Additional insights from this gender-based analysis are illustrated in Figure-3A. Age group comparisons revealed that only bloody diarrhea and ecchymosis exhibited significant differences in distribution (Figure-3B).

**Figure-4:**
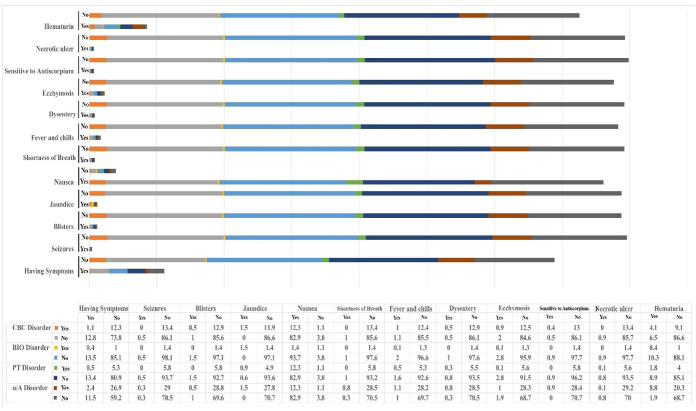
Figure 4. Overall view on clinical manifestations prevalence based on positive and negative history of laboratory disturbance including CBC, BIO, PT, and u/A.

**Figure-5:**
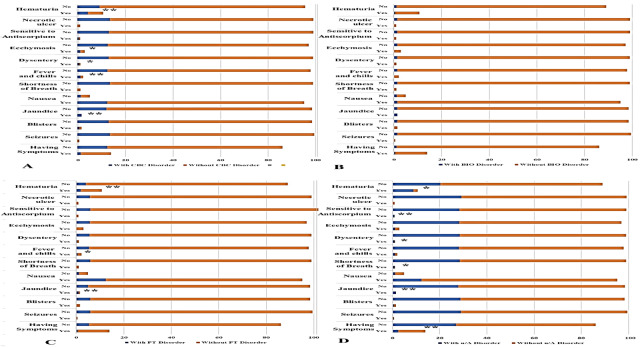
Figure 5. Detailed view on clinical manifestations prevalence based on positive and negative history of laboratory disturbance including CBC (A), BIO (B), PT (C), and u/A (D).*: P-value ≤ 0.01; **: P-value ≤0.001

Moreover, an analysis of patients with delayed hospital visits indicated that the absence of symptoms and vital clinical signs such as blurred vision, dizziness, jaundice, tenderness, necrotic ulcers, and hematuria varied significantly among different groups (Figure-3C). When examining bite site categorization, clinical symptoms, including blurred vision, bloody diarrhea, ecchymosis, necrotic ulcers, and hematuria, presented significant distinctions (Figure-3D). Additionally, a review of symptoms across varying seasons of bites indicated significant differences in blistering, dizziness, jaundice, tenderness, necrotic lesions, and hematuria (Figure-3E). In the context of ICU admissions, significant differences in clinical symptoms such as seizures, blurred vision, dizziness, jaundice, nausea, shortness of breath, bloody diarrhea, muscle spasms, and hematuria were recorded among the various ICU groups (Figure-3F). Furthermore, among different treatment groups receiving the antivenom (Anti-scorpion), significant differences were observed in the absence of symptoms, blisters, jaundice, nausea, shortness of breath, bloody diarrhea, tenderness, sensitivity to antiscorpion, necrotic ulcers, and hematuria (Figure-3G). The treatment groups involving Pack Cells also displayed significant differences in symptoms of blistering, dizziness, jaundice, fever and chills, bloody diarrhea, ecchymosis, and hematuria (Figure-3H). Lastly, a comparative analysis of patient populations categorized by CBC disturbance, BIO disturbance, PT disturbance, and u/A disturbance is presented in Figure-4. In the comparison between CBC disturbance and non-CBC disturbance groups, symptoms such as jaundice, fever and chills, bloody diarrhea, ecchymosis, and hematuria displayed significantly different distributions (Figure-5A). In relation to the BIO variable, no significant differences were found among the clinical symptoms (Figure-5B). However, significant differences regarding jaundice, fever and chills, and hematuria were noted in the different PT disturbance groups (Figure-5C). In the u/A disturbance groups, significant differences emerged in the absence of symptoms and the presence of symptoms such as jaundice, shortness of breath, bloody diarrhea, sensitivity to antiscorpion, and hematuria (Figure-5D).

## Discussion

The scorpionism is an actual public health problem in several parts of the world, particularly in tropical and subtropical regions. The treatment of scorpion envenomation is complex and controversial, in particular regarding the utility of the antivenoms and symptomatic treatments that should be associated [1]. Every year, approximately 1.2 million people are victims of scorpion stings globally, leading to an estimated 3,000 deaths annually, making scorpions the second most deadly venomous creatures after snakes [10]. Scorpion stings represent a significant public health issue in Iran. The Iranian Non-Communicable Diseases Committee (INCDC) reports that approximately 50,000 cases of scorpion stings occur annually across the country [11]. Recent estimates place the average treatment cost for scorpion stings at approximately $1,192 per case, which highlights the financial burden associated with these incidents. This cost, although lower than that of snake bites averaging $2,104 per case, still poses significant challenges for affected individuals and the healthcare system in Iran [12].

The present study aims to explore the relationship between clinical and laboratory symptoms in patients suffering from scorpion stings who were hospitalized at Razi Hospital, Ahvaz, over a span of four years from 2018 to 2022. The findings revealed that 55.9% of the patients were male, and the majority of incidents occurred in individuals aged 21 to 40 years, making up 48.1% of the cases. Notably, the incidence of stings demonstrated a marked increase during the summer months compared to the other seasons. These trends align with findings from Shahrabadi et al., which also identified a higher prevalence among males, particularly in the 30 to 40 age group, and indicated that the majority of stings occur during the summer months [13]. In the research conducted by Bosnak et al. in southeastern Turkey, approximately 75% of the scorpion sting victims were male, with the highest incidence occurring during the summer months, accounting for 78.8% [14]. A case-control study referencing Brazil’s official reporting systems indicated that, during the study period, there were 2,120 reported instances of scorpion stings in the State of Amazonas, predominantly affecting males (63.9%), with the most impacted age group being individuals between 21 and 30 years old (17%) [15].

The noteworthy differences in sting incidence across age groups can be attributed to the activities and lifestyles of individuals that heighten their risk of encountering scorpions. In the summer, both human and animal activities escalate, leading to increased probabilities of encounters. Human interaction with the natural environment tends to be less aggressive in winter months, further mitigating sting occurrences [16]. Moreover, scorpions exhibit less potent venom during the winter, as their venom gland concentration diminishes [17]. The analysis of present study revealed that the lower limbs were the most common sites for stings, followed closely by the upper limbs. The primary clinical symptoms reported were pain, erythema, and swelling at the sting sites. To illustrate, in a cross-sectional study conducted by Shahsavarinia et al. in 2017, where patients with a history of scorpion stings visiting the emergency department of Sina Hospital, Tabriz were assessed, it was found that the upper limbs accounted for the highest number of stings at 47.2%. In assessing the clinical signs and symptoms of the patients, a staggering 89.9% reported pain, while 48.8% experienced localized erythema, 21% exhibited swelling at the sting site, and 0.6% reported respiratory manifestations [18]. Vaucel et al. conducted a retrospective study covering a span of 16 years in 2019, revealing that the hands and feet accounted for 84% of the sting locations [19]. Al-Asmari et al. [20] indicated that local pain was the primary complaint among 95% of patients, a finding also supported by Duru et al., [21] who noted pain as the most common complication in Turkish patients.

This evidence correlates strongly with the current study’s findings, reinforcing the notion that distal limbs, such as hands and feet, exhibit greater risk for scorpion stings due to their heightened exposure to the environment during various activities. The higher density of stings in the upper limbs may be attributed to occupational hazards, where individuals are likely to handle materials in scorpion habitats, such as working in fields or lifting stones and bricks. In contrast, stings in the lower limbs might occur due to a lack of preventive measures, including inadequate footwear at home or in agricultural settings, neglecting to check shoes before wearing them, and walking barefoot.

The results of the present study showed that most individuals had referred to the hospital within the first three hours, the majority of the patients had been hospitalized for at least one day, and also most participants had received one vial of antiscorpion. Jaberhashemi et al. reported in 2023 that 28 percent of individuals had referred to the emergency room within three hours after the sting [22]. In Queiroz et al.’s study, 69.6% of cases sought medical assistance within the first three hours post-sting [15]. In contrast, Bosnak et al. documented a mean hospital arrival time of 5.1 ± 2.8 hours [14]. The reasons for this delay could include distance from the city center, inadequate rural roads, unavailability of transportation, and lack of awareness regarding the importance of receiving treatment as soon as possible. The results of the present study indicated that the most common laboratory disorder was U/A disturbance. The highest amounts of FFP and PLT received were 4 cases of patients (0.5 percent) received 1 FFP, and 2 cases of patients (0.3 percent) received 4 PLT, and 2 cases of patients (0.3 percent) received 6 PLT. In the study by Soleimani and colleagues in 2021, the most common laboratory disorder was also found to be a disturbance in urinalysis [23].

The results of the current study indicate that out of the total patients, 12.3% were hospitalized in the ICU, 2 individuals (0.3%) were intubated, and 2 individuals (0.3%) died. Bosnak et al. noted that all patients except for one child, who tragically succumbed to severe pulmonary edema, recovered after treatment [14]. Mahshidfar and colleagues, in a cross-sectional study conducted in Iran in 2017 in southwestern Iran over the course of one year [24]. The average time to hospital visit was 1.89±1.04 hours, and 5 cases (0.2%) resulted in death. For clinical symptom of sensitivity to anti-scorpion and laboratory findings of BIO disturbance and organ dysfunction, it was a significant difference between male and female groups, because all cases sensitive to anti-scorpion serum were male, while 90% of those with BIO disturbance and 100% of those with organ dysfunction were female.The distribution of clinical and laboratory symptoms among different age groups of the patients indicated a significant relationship between symptoms of bloody diarrhea, ecchymosis, and organ dysfunction with the age of the patients. 50% of bloody diarrhea cases were under 20 years old, and the other 50% were between 21 and 40, indicating the presence of bloody diarrhea at younger ages, while no cases of bloody diarrhea occurred in those over 40 years old. About 65% of ecchymosis cases were under 40 years old, indicating that ecchymosis is more common at younger ages and decreases with age. Approximately 85% of organ dysfunction cases were under 20 years old. In the study by Soleimani and colleagues, the incidence of bloody diarrhea and organ dysfunction in children was observed to be higher [23], which may be due to the sensitivity and lower tolerance levels of younger individuals to scorpion venom, thus indicating the need for greater caution and more control over children’s activities during warm seasons.

There was a significant relationship between clinical symptoms, including blisters, dizziness, jaundice, tenderness, necrotic wounds, burning, hematuria, erythema, pain, laboratory symptoms, including CBC disorders, PT disturbance, and U/A disorders, and the season of the sting. Specifically, 84% of patients with blisters, 84% of patients with jaundice, 72% of patients with necrotic wounds, and 55% of patients with hematuria presented in the spring, while 88% of patients with CBC disorders, 68% of patients with PT disturbance, and 81% of patients with UA disorders presented in the spring and summer. These findings suggest that the prevalence of these symptoms is higher in the spring and summer. The reason for this is that scorpions have just awakened from their winter sleep in the spring, and the scorpion’s venom gland has a higher concentration in the spring and summer.

The relationship between the frequency of clinical symptoms and laboratory signs, with the ICU admissions was significant, such that clinical symptoms such as seizures (100%), jaundice (100%), dyspnea (100%), bloody diarrhea (75%), hematuria (40%), and laboratory signs including CBC disturbance (40%), BIO disturbance (65%), PT disturbance (50%), U/A disturbance (26%), and organ disturbance (100%), were more common in patients admitted to the ICU. Vaucel et al. reported that among scorpion-stung patients, 76% exhibited local symptoms, while 40% showed cardiac symptoms, 15% had gastrointestinal issues, and 12% experienced neurological symptoms; additionally, 35% required hospitalization, with 5% necessitating admission to the ICU, revealing that the severity of symptoms was greater in those admitted to the intensive care unit [19]. These findings are consistent with the results of the present study. The relationship between clinical and laboratory symptoms, and the amount of P.C received was significant, in such a way that most patients with blisters (84%), bloody diarrhea (75%), ecchymosis (91%), hematuria (80%), swelling (100%), CBC disturbance (88%), and UA disturbance (90%) did not require P.C.

However, 100% of patients with jaundice and 36% of patients with BIO disturbance required P.C, indicating the presence of hemolysis and drop in Hb, which itself causes jaundice and also leads to kidney damage. In a study conducted by Mr. Valavi and colleagues in 2016 in Ahvaz, scorpion-stung patients who experienced hemolysis and symptomatic anemia, such as jaundice, hemodynamic failure, and heart failure, received P.C [25]. The present findings demonstrate a significant relationship between clinical symptoms and laboratory results obtained from complete blood counts (CBC). Notably, all patients exhibiting jaundice also presented with a decrease in hemoglobin (Hb) levels, alongside increases in blood urea nitrogen (BUN) and creatinine (Cr).

This pattern can be attributed to the heightened red blood cell (RBC) hemolysis prompted by scorpion venom, which ultimately leads to jaundice via increased bilirubin production. Conversely, among the patients who did not exhibit a significant drop in Hb levels, a substantial 87% did not have jaundice. This observation supports the conclusion that unless hemolysis occurs resulting in a decrease in Hb, patients are unlikely to present with jaundice.

While our study comprehensively analyzed all accessible data for each patient, several limitations warrant consideration. The assessment of treatment outcomes solely at the time of discharge, without subsequent follow-up, introduces potential inaccuracies regarding long-term effectiveness. The presence of missing data further compromises the accuracy of our conclusions and may introduce bias. Additionally, the absence of current and comprehensive national data on scorpion stings hinders a broader understanding of the issue within the country. The retrospective nature of the study, relying on pre-existing medical records, poses challenges related to data consistency and standardization across different healthcare providers. Variations in diagnostic criteria, treatment protocols, and documentation practices could introduce significant heterogeneity in the data, affecting the reliability of our findings. Finally, the study’s cross-sectional design precludes the establishment of causal relationships between specific interventions and patient outcomes; future longitudinal studies are needed to evaluate the long-term efficacy of different treatment strategies.

## Conclusion

The findings of the present study elucidate a concerning trend in scorpion sting incidents, revealing that 55.9% of affected patients were male, with the highest incidence occurring among individuals aged 21 to 40 years. Notably, summer emerged as the peak season for these encounters, underscoring the seasonal nature of this public health issue. The data indicated that most sting sites were located on the lower limbs, followed closely by the upper limbs, with pain, erythema, and swelling being the most prevalent clinical symptoms reported.

Disturbance in U/A results were identified as the most common laboratory disorder, while the association of clinical symptoms with laboratory disturbances was more pronounced among women, younger patients, those admitted to intensive care units, and individuals who presented within the first hour following a sting. Of particular note is jaundice, which, despite appearing later than other symptoms, remained the most common clinical sign linked to abnormal laboratory findings. By corroborating these results with existing literature, this five-year evaluation highlights the multifaceted challenges posed by scorpion stings, which not only impact patient health but also impose a substantial financial burden on families and the healthcare system. By prioritizing community education and equipping healthcare systems with robust training protocols, we can significantly mitigate the risks and consequences of scorpion stings, ultimately safeguarding public health and well-being in vulnerable populations.

## Conflict of Interest

The authors have no conflicts of interest to declare.

## References

[R1] Rahmani A, Forouzandeh H, Kalantar M, Asad-Masjedi N, Alavian Z, Kavarizadeh K (2015). Epidemiological and clinical characteristics of scorpion stings
in Ahwaz, Southwest Iran (2006-2010). Int J Med Toxicol Forensic Med.

[R2] Khanbashi S, Khodadadi A, Assarehzadegan MA, Pipelzadeh MH, Vazirianzadeh B, Hosseinzadeh M, et al (2015). Assessment of immunogenic characteristics of Hemiscorpius
lepturus venom and its cross-reactivity with venoms from Androctonus
crassicauda and Mesobuthus eupeus. J Immunotoxicol.

[R3] Rahmani A, Jalali A (2012). Symptom patterns in adult patients stung by scorpions with
emphasis on coagulopathy and hemoglubinuria. J Venom Anim Toxins Incl Trop Dis.

[R4] Bavani MM, Saeedi S, Saghafipour A (2021). Spatial distribution of medically important scorpions in Iran: A
review article. Shiraz E Med J.

[R5] Hussen F, Erdek M, Yağmur E (2023). External morphology of Hemiscorpius lepturus Peters, 1861
(Scorpiones: Hemiscorpiidae). Arthropoda Sel.

[R6] Jalali A, Pipelzadeh M, Seyedian R, Rahmani A, Omidian N (2011). In vivo pharmacological study on the effectiveness of available
polyclonal antivenom against Hemiscorpius lepturus venom. J Venom Anim Toxins Incl Trop Dis.

[R7] Dehesa-Davila M, Alagon AC, Possani LD (221-38).

[R8] Khattabi A, Soulaymani-Bencheikh R, Achour S, Salmi L-R (2011). Classification of clinical consequences of scorpion stings:
consensus development. Trans R Soc Trop Med Hyg.

[R9] https://nursingdabadanumsacir/portal/home/?288112/.

[R10] Boubekeur K, L’Hadj M, Selmane S (2020). Demographic and epidemiological characteristics of scorpion
envenomation and daily forecasting of scorpion sting counts in Touggourt,
Algeria. Epidemiol Health.

[R11] Mousavi SA, Rashidi H, Faramarzi A, Feyzi R, Kaidkhordeh M, Fard PF (2023). Epidemiology of Scorpion Sting in Southwestern Iran Over Five
Years. Trends med sci.

[R12] Mashhadi I, Kavousi Z, Peymani P, Ramhormozi SSZ, Keshavarz K (2017). Economic burden of scorpion sting and snake bite from a social
perspective in Iran. Shiraz E Med J.

[R13] Shahrabadi E, Moradi M, Rezaeian M, Salimabadi Y, Esmaeili Ranjbar, Moinaddini S (2020). The epidemiological study of clinical signs and outcomes of
patients with scorpion stings referred to emergency department of Rafsanjan
Ali-Ibn-Abitaleb Hospital in 2017-2018: a descriptive study. J Rafsanjan Univ Med Sci.

[R14] Bosnak M, Ece A, Yolbas I, Bosnak V, Kaplan M, Gurkan FJW (2009). Scorpion sting envenomation in children in southeast Turkey. Wilderness & environmental medicine.

[R15] Queiroz AM, Sampaio VS, Mendonça I, Fé NF, Sachett J, Ferreira LCL (2015). Severity of scorpion stings in the Western Brazilian Amazon: a
case-control study. PLoS One.

[R16] Polis GA (1980). Seasonal patterns and age-specific variation in the surface
activity of a population of desert scorpions in relation to environmental
factors. J Anim Ecol.

[R17] Tobassum S, Tahir HM, Zahid MT, Gardner QA, Ahsan MM (2018). Effect of milking method, diet, and temperature on venom
production in scorpions. J Insect Sci.

[R18] Shahsavarinia K, Taghizadieh A, Ghaffarzad A, Shariati A, Rahmani F (2017). Epidemiological and clinical status of patients with scorpion
sting: emergency department of Sina hospital in Tabriz-Iran. J emerg pract trauma.

[R19] Vaucel J, Labadie M, Hoarau M, Kallel H (2019). Pediatric scorpionism in French Guiana: Epidemiological and
clinical study–Preliminary result. Ann Toxicol Anal.

[R20] Al-Asmari AK, Al-Saif AAJSmj (2004). Scorpion sting syndrome in a general hospital in Saudi Arabia. Saudi medical journal.

[R21] Duru M, Karakuş A, Yengil E, Sahan M, Zeren C, Kekec Z Demographic, clinic and laboratory characteristics of cases
presented to emergency department with scorpion sting: 345 cases over a
4-years period. Acta Medica Mediterranea.

[R22] Jaberhashemi SA, Amiri Z, Norouzi M, Shahi M (2023). Epidemiological Factors and Indicators Related to Venomous Bites
and Stings in High-risk Areas of Southern Iran. J Prev Med.

[R23] Soleimani G, Shafighi Shahri, Shahraki N, Godarzi F, Soleimanzadeh Mousavi, Tavakolikia Z (2021). Clinical and Laboratory Findings and Prognosis of Snake and
Scorpion Bites in Children under 18 Years of Age in Southern Iran in 2018-19. Int J Pediatr.

[R24] Mahshidfar B, Ghafouri HB, Yasinzadeh MR, Mofidi M, Rezai M, Farsi D (2017). Demographics of scorpion sting in Iran; a cross sectional study. Emergency.

[R25] Valavi E, Amuri P, Ahmadzadeh A, Ahankoob E (2016). Acute kidney injury in Hemiscorpius lepturus scorpion stung
children: risk factors and clinical features. Saudi J Kidney Dis Transpl.

